# Deep learning-based measurement of split glomerular filtration rate with ^99m^Tc-diethylenetriamine pentaacetic acid renal scan

**DOI:** 10.1186/s40658-024-00664-w

**Published:** 2024-07-17

**Authors:** Sejin Ha, Byung Soo Park, Sangwon Han, Jungsu S. Oh, Sun Young Chae, Jae Seung Kim, Dae Hyuk Moon

**Affiliations:** 1 Department of Nuclear Medicine, Asan Medical Center, University of Ulsan College of Medicine, 88 Olympic-ro 43-gil, Songpa-gu, Seoul, 05505 Republic of Korea; 2 Department of Nuclear Medicine, Uijeongbu Eulji Medical Center, Eulji University School of Medicine, Uijeongbu, 05505 Republic of Korea; 3 Present Address: Department of Nuclear Medicine, Korea University Anam Hospital, Korea University College of Medicine, Seongbuk-gu, Seoul, 02841 Republic of Korea

**Keywords:** [^99m^Tc]Tc-diethylenetriamine pentaacetic acid, Glomerular filtration rate, Deep learning, Convolutional neural networks, Image segmentation

## Abstract

**Purpose:**

To develop a deep learning (DL) model for generating automated regions of interest (ROIs) on ^99m^Tc-diethylenetriamine pentaacetic acid (DTPA) renal scans for glomerular filtration rate (GFR) measurement.

**Methods:**

Manually-drawn ROIs retrieved from a Picture Archiving and Communications System were used as ground-truth (GT) labels. A two-dimensional U-Net convolutional neural network architecture with multichannel input was trained to generate DL ROIs. The agreement between GFR values from GT and DL ROIs was evaluated using Lin’s concordance correlation coefficient (CCC) and slope coefficients for linear regression analyses. Bias and 95% limits of agreement (LOA) were assessed using Bland-Altman plots.

**Results:**

A total of 24,364 scans (12,822 patients) were included. Excellent concordance between GT and DL GFR was found for left (CCC 0.982, 95% confidence interval [CI] 0.981–0.982; slope 1.004, 95% CI 1.003–1.004), right (CCC 0.969, 95% CI 0.968–0.969; slope 0.954, 95% CI 0.953–0.955) and both kidneys (CCC 0.978, 95% CI 0.978–0.979; slope 0.979, 95% CI 0.978–0.979). Bland-Altman analysis revealed minimal bias between GT and DL GFR, with mean differences of − 0.2 (95% LOA − 4.4–4.0), 1.4 (95% LOA − 3.5–6.3) and 1.2 (95% LOA − 6.5–8.8) mL/min/1.73 m² for left, right and both kidneys, respectively. Notably, 19,960 scans (81.9%) showed an absolute difference in GFR of less than 5 mL/min/1.73 m².

**Conclusion:**

Our DL model exhibited excellent performance in the generation of ROIs on ^99m^Tc-DTPA renal scans. This automated approach could potentially reduce manual effort and enhance the precision of GFR measurement in clinical practice.

**Supplementary Information:**

The online version contains supplementary material available at 10.1186/s40658-024-00664-w.

## Introduction

Accurate measurement of GFR is essential for diagnosing and managing renal diseases, adjusting medication dosages and monitoring the progression of kidney dysfunction in various clinical settings. ^99m^Tc-diethylenetriamine pentaacetic acid (^99m^Tc-DTPA) is the most widely used radiopharmaceutical for measuring glomerular filtration rate (GFR). It has little in the way of protein binding properties and is excreted by glomerular filtration without renal tubular secretion [1]. Split renal GFR is the most important quantitative marker derived from radionuclide renography with ^99m^Tc-DTPA and is crucial for clinical decision making in many clinical situations, including hydronephrosis, vesicoureteral reflux, renal artery stenosis, tumourous conditions, and renal transplant donor evaluation [2].

A commonly used method to calculate GFR using a gamma camera involves the Gates formula, which requires regions of interest (ROIs) to be set for both kidneys and their background area [3, 4]. Because only 20% of renal blood flow is generally filtered by the glomeruli [5], ^99m^Tc-DTPA images show relatively low signal-to-noise ratio with low renal uptake and high background activity. Therefore, GFR measurement by the Gates method might be sensitive to the ROI settings [6]. GFR calculation on ^99m^Tc-DTPA scans is usually performed using commercially available software that provides manual or semi-automated tools with a master ROI and a subsequent thresholding for drawing ROIs; the choice of how to set ROIs can vary depending on the operator [7]. Operator dependency in setting ROIs limits the accuracy and reproducibility of GFR measurements and can adversely affect clinical decision-making.

Artificial intelligence based on convolutional neural network (CNN) algorithms is increasingly being applied in nuclear medicine imaging analysis [8–14]. In early studies, U-net-based deep learning (DL) algorithms have shown favorable results in image segmentation tasks such as tumour delineation on PET/CT scans. Such deep learning-based image segmentation can be trained particularly well when large amounts of data with well-controlled labels are available, and has the potential to be applied in routine clinical practice to improve the accuracy and reproducibility of GFR measurement on ^99m^Tc-DTPA scans, reducing manual effort and time.

Therefore, we propose a two-dimensional (2D)-CNN model for the automatic generation of kidney ROIs, which we trained with extensive datasets of ^99m^Tc-DTPA scans with labelled ROI data from structured report forms retrieved from our Picture Archiving and Communication System (PACS).

## Materials and methods

### Patients

This is a single-center study involving data from patients who underwent a DTPA renal scan for the measurement of split GFR at Asan Medical Center (Seoul, Korea) between October 2009 and November 2021. Scans were excluded from the analysis because of any one of the following: (1) presence of a single kidney (i.e., post-nephrectomy state or transplant kidney); (2) severely decreased split renal function of less than 20% [15]; (3) inconsistency in the number of frames (not 80); and (4) patient age of ≤ 15 years [16]. This study was conducted in accordance with the Declaration of Helsinki and our institutional guidelines. Our local institutional review board approved this study (IRB No.2022 − 0333) and waived the need to obtain informed consent because of its retrospective nature.

Electronic medical records and ^99m^Tc-DTPA scans were retrospectively reviewed by the authors. Serum creatinine, blood urea nitrogen (BUN) level and GFR estimated by the CKD-EPI equation [17] within 1 month from scan were obtained.

### DTPA scan acquisition and report

Quantitative ^99m^Tc-DTPA renal scans were performed as previously described [18]. Shortly, dual-head gamma cameras used for imaging (Intevo Bold, Intevo 16, Symbia E, Symbia T2, E.cam, Evo Excel; Siemens Healthineers, Erlangen, Germany) after intravenous injection of 185 MBq of ^99m^Tc-DTPA. Image acquisition was initially performed with low-energy high-resolution collimators, then underwent a transition to low-energy all-purpose collimators on July 22, 2014. Dynamic images were captured using a 64 × 64 matrix, with a zoom factor of 1.45, with 15 s per frame over a duration of 20 min, resulting in 80 frames. The exact injection activity was determined by measuring the radioactivity of the syringe before and after injection, taking into account decay correction.

Images were prospectively analysed at the time of image acquisition by expert nuclear medicine radiologic technologists using Syngo workstation (Siemens Healthineers). ROIs for each kidney were manually delineated. If tumours were present, they were excluded from the ROI. The perirenal background ROI was set inferolaterally to the center of the kidney, ensuring it was placed 1–2 pixels away from organs to avoid scatter activity.

GFR was calculated on the basis of renal uptake of ^99m^Tc-DTPA measured during a 2–3 min interval (9–12th frame) using a gamma camera-based Gates method [3, 4]. Kidney depth was calculated using the Tonnesen formula [19] until July 2014, then afterwards the Taylor formula [20] was applied on the basis of a previous report [21]. For patients with renal anomalies or variations in kidney position, kidney depth was manually calculated on CT images. The final structured reports were approved by experienced nuclear medicine physicians (S.H., S.Y.C. and D.H.M.) and uploaded to the in-house PACS termed PetaVision.

### Parsing PACS data

Sets consisting of a structured report (1132 × 860 matrix) and 80 raw frame images were acquired in the Digital Imaging and Communications in Medicine (DICOM) file format. In-house software was then used to automatically anonymise the images. Parameters including kidney and background ROIs, time-activity curves, patient age, height, weight, split kidney functions (%), depth and background-adjusted kidney counts (counts per minute), kidney depth (cm), GFR (ml/min) and normalised GFR by body surface area (ml/min/1.73 cm²) were extracted from the structured reports using a character recognition technique involving a simple convolution using a matched filter for printed characters [22]. To elaborate on the method for extracting digits, we extracted each digit (0–9) from the values presented in the report format. We then applied a matched filter approach, utilising patches from the areas where the digits were located. The correlation between these patches and the extracted digits (0–9) was assessed to verify the validity of the digit extraction process.

**Fig. 1:**
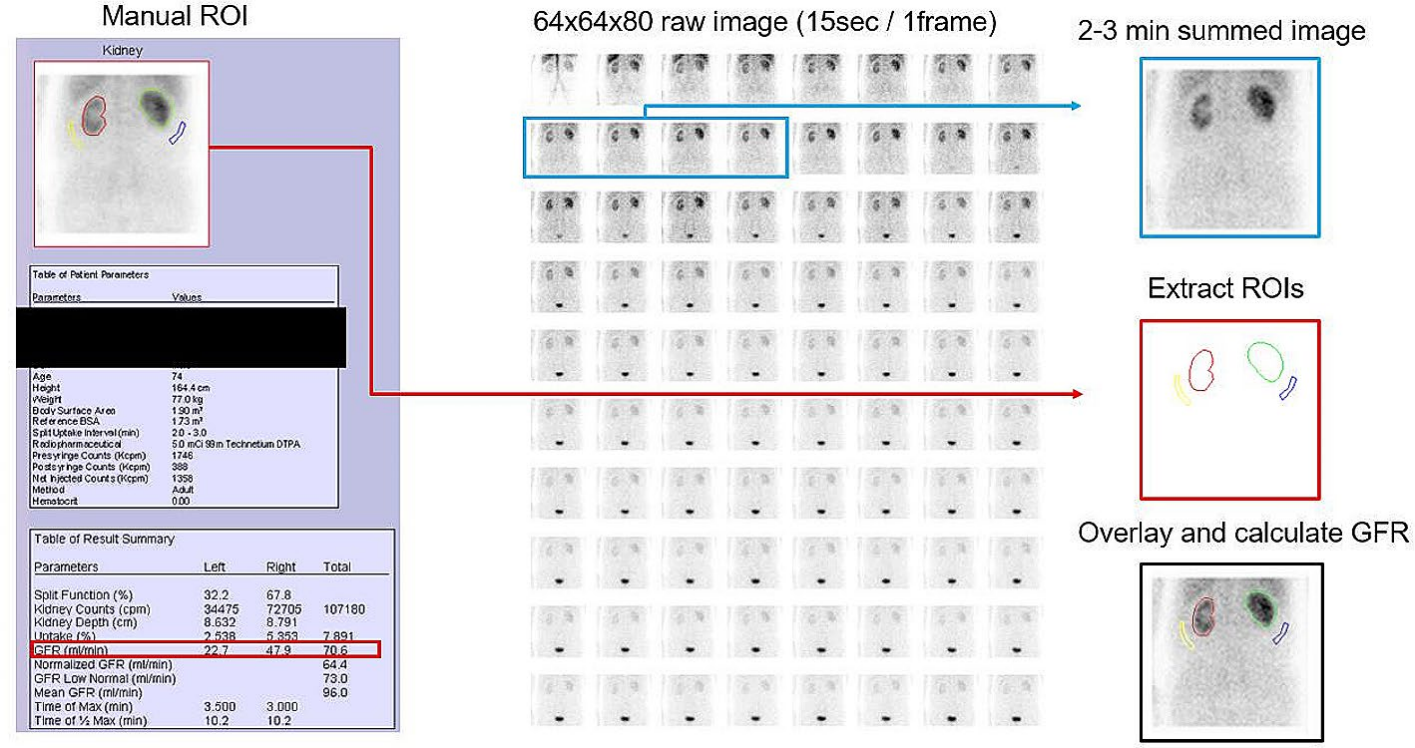
Schematic representation of the manual ROI extraction and GFR calculation process utilised in this study. A structured report along with 80 raw frame images (15 s per frame) was obtained in DICOM format. The images were anonymised automatically with a black-filled square using in-house software. GFR was calculated on the basis of renal uptake of ^99m^Tc-DTPA captured in a 2–3-minute (9–12th frame) summed image (blue arrow). The manual ROIs for each kidney and the background delineated in the structured reports were identified using colour-based edge detection methods (red arrow). These ROIs were subsequently superimposed onto the 2–3-minute summed images for GFR estimation

Manual ROIs of each kidney and background within the structured report were extracted using colour-based edge detection techniques, and were then overlaid onto the 2–3-minute summed images to estimate GFR (Fig. 1). To confirm successful extraction, we compared the kidney counts for each kidney in the structured report with those obtained from the extraction process. The report format depicted ROIs as contours (red/green for kidneys, yellow/blue for backgrounds) rather than filled areas. Typically, these contours formed a closed circuit, providing contiguous pixel connections. In cases of open contours, we applied morphological operations in Python, including binary closing and hole filling, to ensure the integrity of the ROIs. Scaling of ROI dimensions from the report format image space (192 × 192 or 166 × 166) to the raw data space (64 × 64) was performed before the overlay onto raw data.

### DL training

**Fig. 2:**
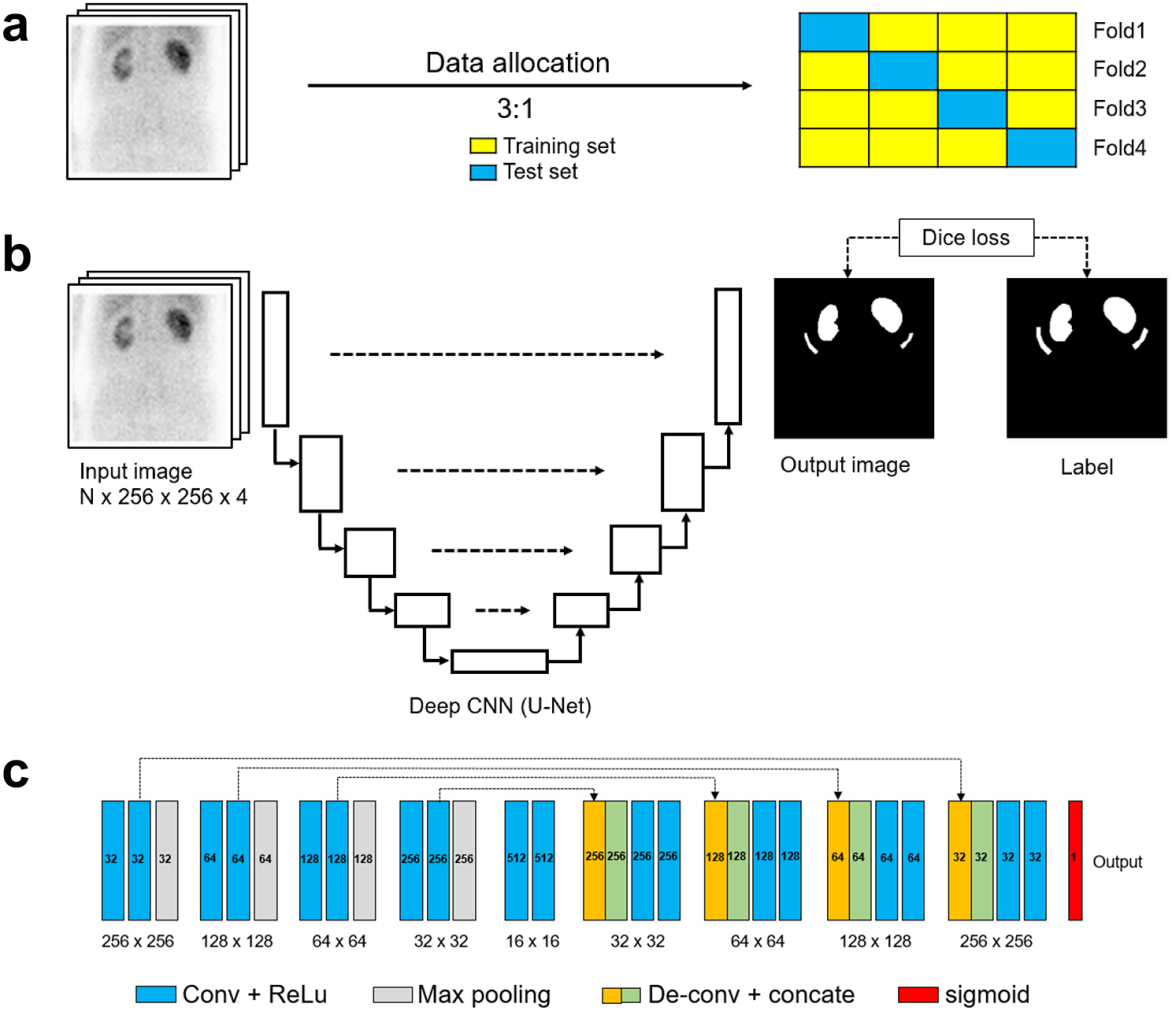
Schematic diagrams illustrating the data allocation and deep CNN architecture (**a**) Data allocation strategy (training: test-set ratio of 3:1) with a four-fold cross-validation-like training scheme. (**b**) U-Net architecture for kidney ROI delineation using deep learning with multi-channel image input (*N* × 256 × 256 × 4). The output image is assessed against the ground truth using the Dice loss function. (**c**) The specific architecture of the U-Net for ROI delineation

Schematic diagrams detailing the setup of the dataset and the structure of the DL model can be found in Fig. 2. Ground-truth (GT) labels were established using the ROIs extracted from each kidney and the background. A set of images (summed over 2–3 min with a resolution of 256 × 256 pixels) and their associated GT ROIs were randomly divided into training (75%) and testing (25%) groups. To assess the model’s efficacy and avoid the risk of overfitting, a four-fold cross-validation-like rotation was executed, yielding four independent test groups, each comprising 25% of the data, with each test group being distinct from the training data [11, 23]. The model’s overall performance was determined by aggregating the results from all four test folds.

A 2D U-Net architecture was designed to accurately replicate the GT ROIs. This U-Net model features a continuous CNN structure that alternates between contracting and expanding paths, integrating residual learning via skip connections for the creation of segmented images. To enhance the model’s ability to generalise, data augmentation techniques such as random translations, rotations, scaling and shearing were applied to both the input and the labels. The Dice similarity coefficient (DSC) served as the loss function, with the adaptive moment estimation algorithm used for optimisation. The model employed rectified linear unit activation functions in its convolutional layers and a sigmoid function for the final activation function to produce binary ROI outputs. Training commenced with an initial learning rate of 1e^‑5^, utilising Tensorflow-based Python scripts (version 2.6.0) on a system equipped with a GeForce NVIDIA RTX 3090 GPU and an AMD Ryzen Threadrippers 3960X CPU. After deep-learning training, a post-processing step was applied to refine the generated masks, employing a 2D-connected component analysis-based kill-islands and fill-holes method.

Significant variability was noted in the spatial and morphological characteristics of the GT background ROIs. To address this, an automated algorithm was developed to generate background ROIs in instances where DL-based background ROIs were not produced. This algorithm performed morphological operations such as dilation, subtraction and angle selection on each 2D kidney mask to create pie-shaped background ROIs positioned at the 5 and 7 o’clock directions for the right and left kidneys, respectively.

### Statistical analysis

Continuous data are presented as either mean ± standard deviation or median with interquartile range (IQR). Lin’s concordance correlation coefficients (CCCs) and slope coefficients for linear regression analyses were used to evaluate the agreements between: (1) kidney counts on the standard PACS report form and those obtained from the extracted GT ROIs (to check the ROI extraction process); and (2) between the GFR values derived from GT ROIs and those derived from DL ROIs (to assess the performance of the DL models). Bland-Altman plots were used to visually assess potential bias and 95% limits of agreement (LOA) between these variables. To facilitate a straightforward interpretation, we evaluated the fraction of scans with an absolute difference between GT and DL GFR of less than 5% or a relative (percent) difference to GT GFR of less than 10%, in which the thresholds were arbitrarily selected. Statistical analyses were performed using R (version 4.1.1; R Foundation for Statistical Computing, Vienna, Austria) and Python (version 3.8; Python Software Foundation, Wilmington, DE).

## Results

### Baseline characteristics

**Fig. 3:**
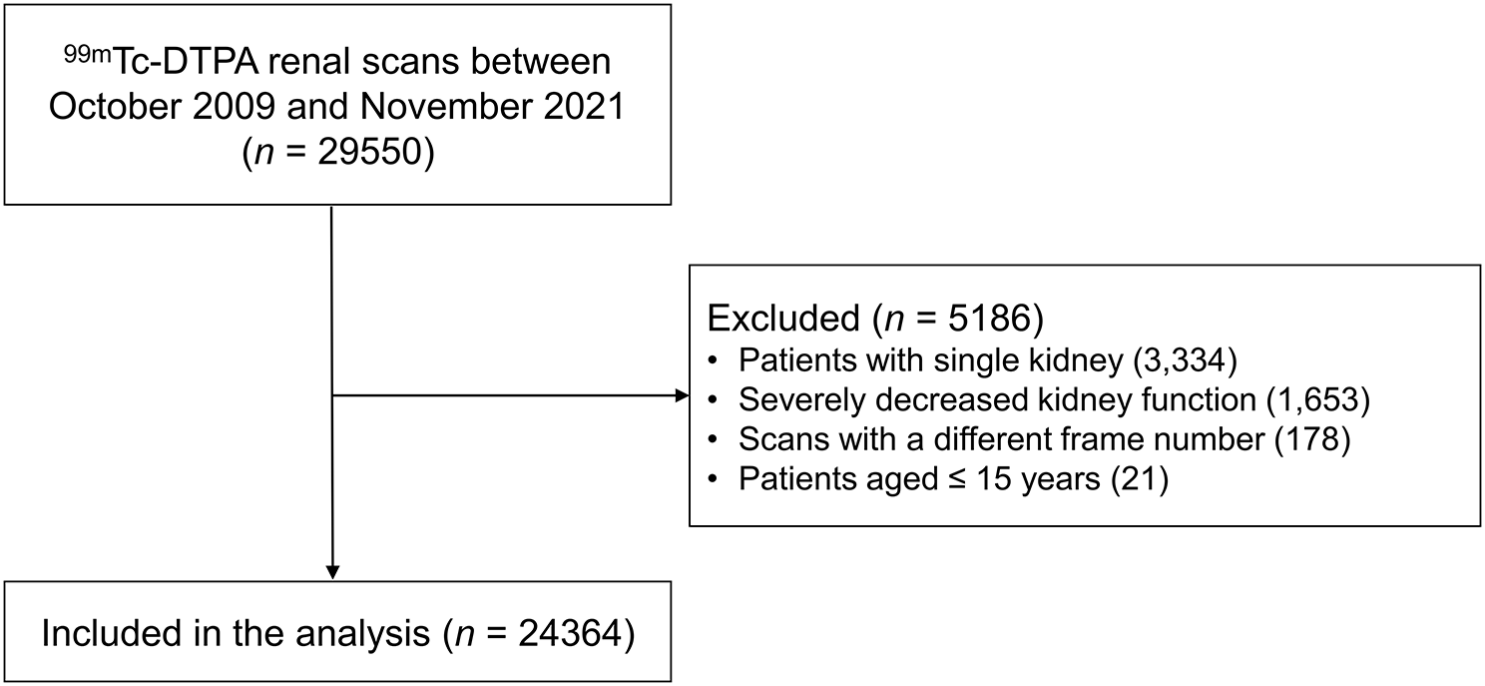
Patient flow diagram

Of 29,550 scans performed during the study period, 3334 were excluded because of the presence of a single kidney, 1653 because of severely decreased kidney function, 178 because of a different number of slices (not 80) and 21 because of patient age ≤ 15 years. These exclusions were made prior to the data acquisition and ROI extraction process. Finally, 24,364 scans from 12,822 patients (male: female = 7794:5028) were included in the DL process and analysis (Fig. 3). The number of scans performed using each gamma camera is summarised in Supplementary Table 1. ^99m^Tc-DTPA scan sessions per patient are summarised in Supplementary Table 2.

The median patient age at the time of scan was 57.0 years (IQR, 47.0–65.0), the median creatinine level was 0.87 (IQR, 0.73–1.01), the median BUN level was 14.0 (IQR, 12.0–18.0) and the median GFR estimated by the CKD-EPI equation was 91.0 (IQR, 77.0–101.0). A total of 9835 DTPA scans were performed on patients following partial nephrectomy. Serum creatinine, BUN, and estimated GFR (by the CKD-EPI equation) levels within one month from the date of ^99m^Tc-DTPA scan were available in 23,260, 12,777, and 17,284 scans, with median time intervals of 0 (IQR 0–2), 0 (IQR 0–1), and 0 (IQR 0–5) days, respectively. These baseline characteristics of the patients are summarized in Table 1.

**Table 1:** Study characteristics

Variables	Total scans	Scans with renal mass	Scans without renal mass
Number of scans	24,364	4067	20,297
Number of patients	12,822	3886	10,741
Age at scan time (years)	57 (47–65)	57 (47–65)	57 (47–65)
Male: Female	15799:8565	2562:1505	13237:7060
History of partial nephrectomy	10,525 (43%)	122 (3%)	10,403 (52%)
Serum creatinine (mg/dL)	0.87 (IQR, 0.73–1.01)	0.86 (IQR, 0.71-1)	0.87 (IQR, 0.71–0.96)
BUN (mg/dL)	14.0 (IQR, 12.0–18.0)	14.0 (IQR, 11.0–17.0)	13.0 (IQR, 11.0–15.0)
Estimated GFR (mL/min/1.73 m^2^)^*^			
< 15	40 (0%)	4 (0%)	36 (0%)
15–30	168 (1%)	19 (0%)	149 (1%)
30–45	519 (2%)	80 (2%)	439 (2%)
45–60	1564 (6%)	271 (7%)	1293 (6%)
60–90	9161 (38%)	1553 (38%)	7608 (37%)
≥ 90	11,451 (47%)	1941 (48%)	9510 (47%)
Not available	1461 (6%)	199 (5%)	1262 (6%)

Data are expressed as number (proportion) or median (interquartile range [IQR])

^*^The estimated GFR was calculated by CKD-EPI equation

### ROI extraction

**Fig. 4:**
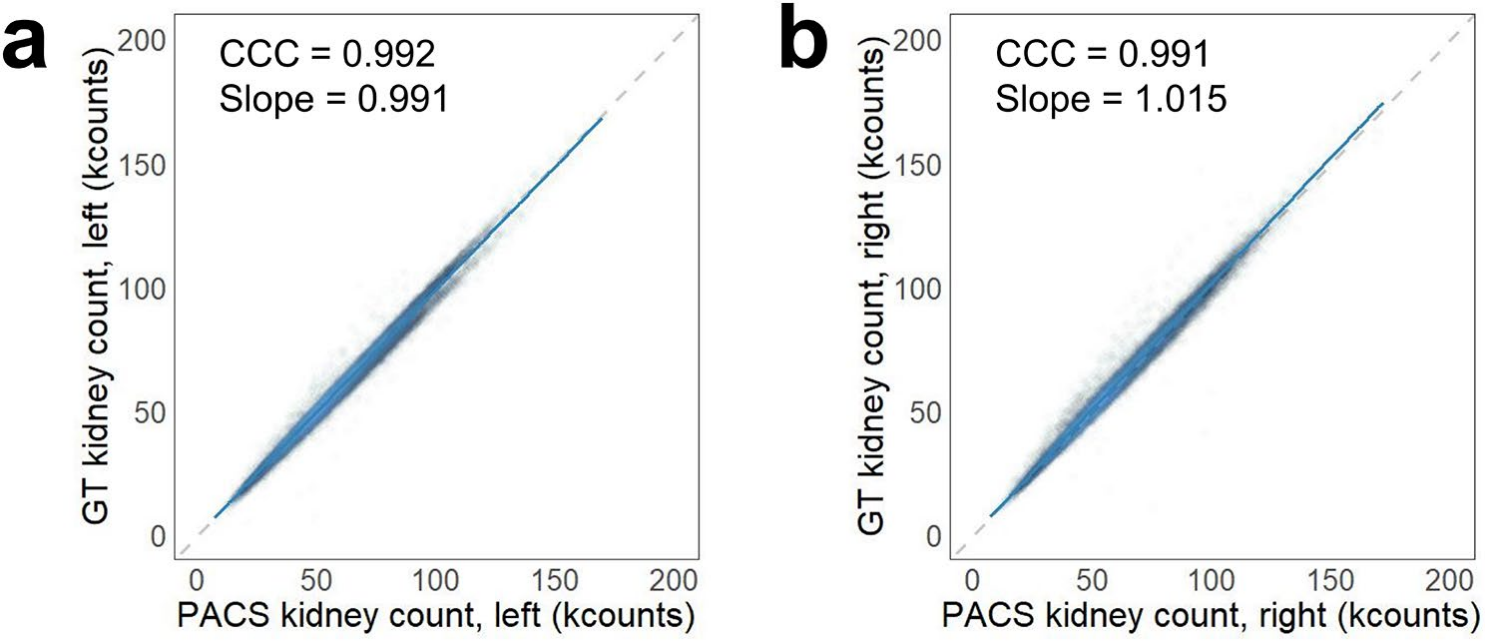
Scatter plots showing the agreement between the counts on the reported PACS form and the count calculated from the extracted ground-truth ROI for (**a**) left kidney and (**b**) right kidney. Dashed lines represent lines of equality

The kidney and background ROIs on the PACS standard report forms were successfully extracted and showed excellent agreement with left kidney counts (CCC 0.992, 95% confidence interval [CI] 0.992–0.993; slope 0.991, 95% CI 0.990–0.991) and right kidney counts (CCC 0.991, 95% CI 0.99–0.991; slope 1.015, 95% CI 1.014–1.016) given in the standard PACS report forms and calculated from the extracted GT ROIs (Fig. 4).

### DL performance evaluation

The mean DSCs across all test sets were 0.928 (95% CI: 0.888–0.968), 0.927 (95% CI: 0.884–0.970) and 0.565 (95% CI: 0.563–0.567) for the left kidney, right kidney and background ROIs, respectively. Of note, our DL model failed to delineate the kidney ROI in four cases. Additionally, in eight cases, DL ROIs for the kidney were substantially different from GT ROIs, with DSC being less than 0.2. The DL model failed to generate a background ROI in 428 scans, and therefore the automatic algorithm-based background ROIs were applied. In the remaining 23,936 cases where DL generated background ROIs, the algorithm-based background ROIs showed excellent agreement with DL ROIs in calculating background count (CCC: 0.962 [95% CI: 0.961–0.963] for the left side, 0.964 [95% CI: 0.963–0.964] for the right side) as well as GFR values (CCC: 0.964 [95% CI: 0.963–0.965] for the left kidney, 0.948 [95% CI: 0.947–0.949] for the right kidney).

**Fig. 5:**
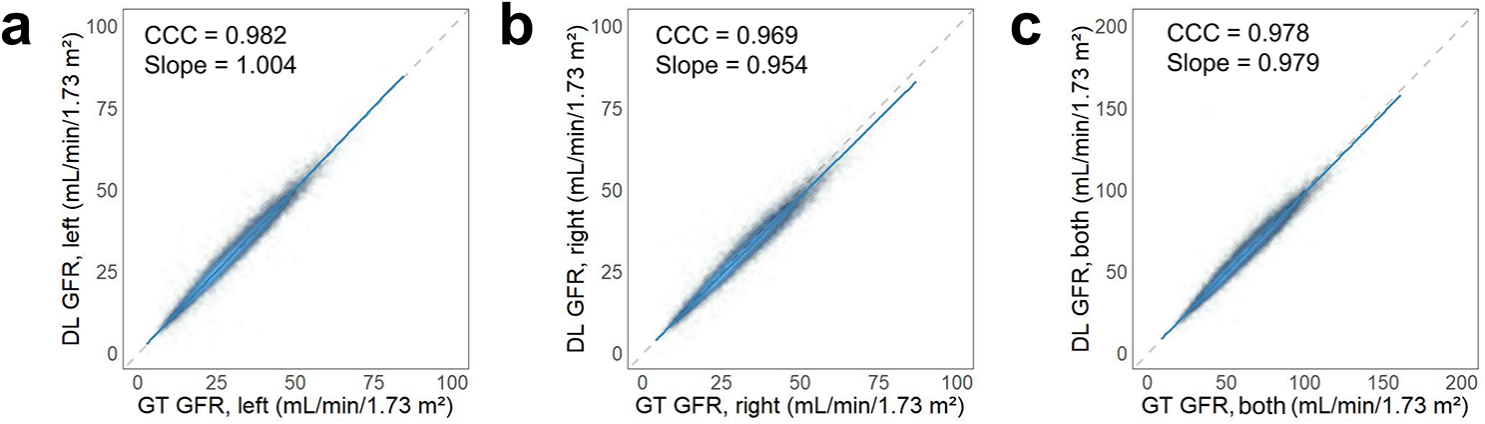
Scatter plots showing the agreement between ground truth (GT) GFR and deep learning (DL) GFR for (**a**) left kidney, (**b**) right kidney and (**c**) both kidneys. Dashed lines represent lines of equality

There was excellent concordance between the GFRs of left kidney (CCC 0.982, 95% CI 0.981–0.982; slope 1.004, 95% CI 1.003–1.004), right kidney (CCC 0.969, 95% CI 0.968–0.969; slope 0.954, 95% CI 0.953–0.955) and both kidneys (CCC 0.978, 95% CI 0.978–0.979; slope 0.979, 95% CI 0.978–0.979) derived from GT and DL ROIs (Fig. 5).

**Fig. 6:**
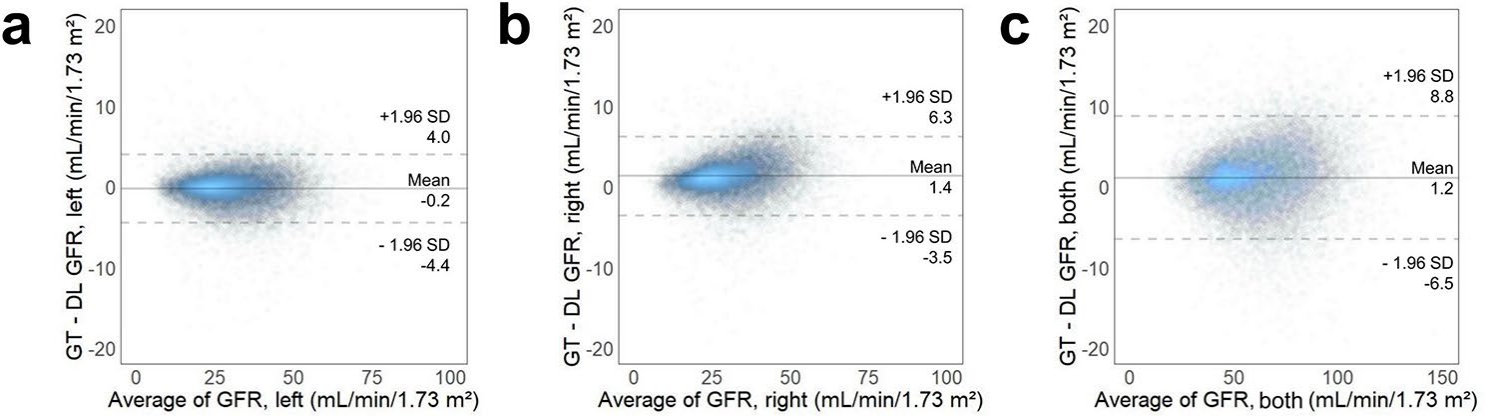
Bland-Altman plots illustrating the agreement between ground truth (GT) GFR and deep learning (DL) GFR for (**a**) left kidney, (**b**) right kidney and (**c**) both kidneys. Solid lines represent mean differences. Dashed lines represent 95% limits of agreement

**Fig. 7:**
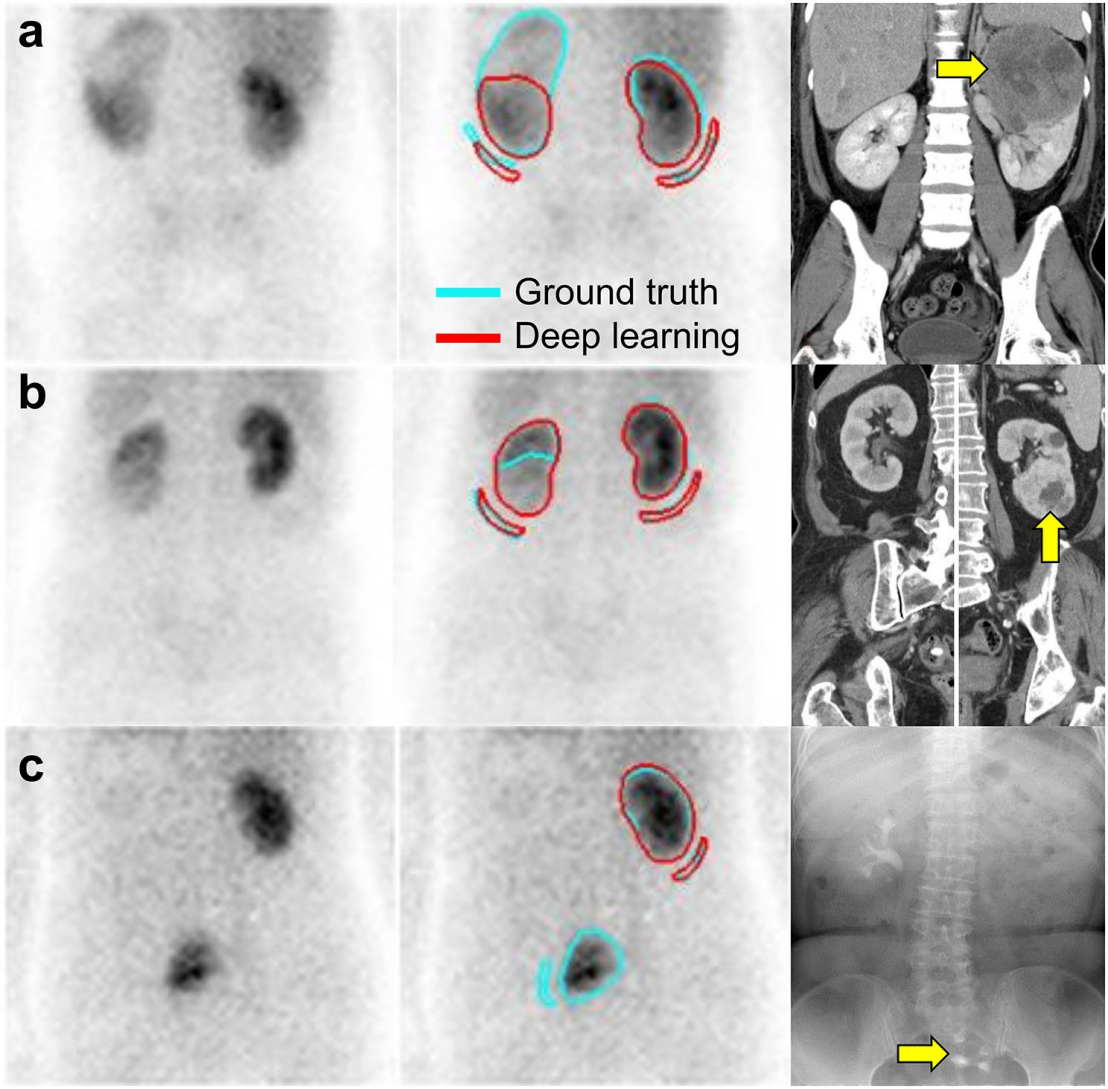
Representative images showing a low concordance between ground truth (GT) ROIs and deep learning (DL) ROIs. Each row displays a 2–3-minute summed image (left), the same image with superimposed ROIs (middle) and a corresponding CT image or intravenous pyelogram (right). Panel (**a**) shows an example where the GT ROI includes a tumour in the upper pole of the left kidney (yellow arrow), whereas the DL ROI does not. Panel (**b**) shows a case where the GT ROI excludes the tumour in the lower pole of left kidney (yellow arrow), but the DL ROI includes it. Panel (**c**) presents a left ectopic kidney in the pelvic cavity that was not delineated by the DL ROI (yellow arrow)

The Bland-Altman analysis showed mean differences between GFR from GT and DL ROIs of − 0.2 (95% LOA: −4.4 to 4.0), 1.4 (95% LOA: −3.5 to 6.3) and 1.2 (95% LOA: −6.5 to 8.8) mL/min/1.73 m^2^ for the left, right and both kidneys, respectively (Fig. 6). Scatter plots and Bland-Altman plots for each test fold are presented in Supplementary Figs. 1 and 2. For 19,960 scans (81.9%), the absolute difference between the GFR from GT and DL ROIs in both kidneys was less than 5 mL/min/1.73 m^2^. The total GFR values showed a percent difference of less than 10% between the DL ROI and GT ROI methods in 22,023 (90.4%) cases, and exhibited a percent difference of less than 20% in 24 133 (99.1%) cases. Representative cases where DL failed to draw an ROI or showed a low concordance compared with GT are presented in Fig. 7. Upon visual review, poor renal uptake, severe hydronephrosis, large renal tumour, or an ectopic kidney was often accompanied in the cases where kidney ROI was not drawn or had a low concordance.

Regarding the presence of renal mass, the excellent agreement was observed in scans without renal mass (CCC 0.979, 95% CI 0.979–0.980; slope 0.979, 95% CI 0.978–0.979) and in those with renal mass (CCC 0.973, 95% CI 0.971–0.974; slope 0.980, 95% CI 0.978–0.982). Scatter plots and Bland-Altman plots according to the presence of renal mass are shown in Supplementary Fig. 3.

## Discussion

Our study demonstrated the accuracy of a 2D-CNN with a U-Net structure for delineating kidney ROIs and measuring GFR on ^99m^Tc-DTPA renal scans. The GFR values obtained from DL-based ROIs were in close agreement with those from manually-drawn ROIs, showing an absolute difference of less than 5 mL/min/1.73 m^2^ in 81.9% of cases, and a relative difference within 10% in 90.4% of cases. These results suggest that using our DL model, 80–90% of patients may complete the GFR measurement without additional effort required for manual correction. Even in the remaining 10–20% of cases, editing of the ROI initially generated by the DL, instead of doing everything manually, could reduce the overall effort for analysing ^99m^Tc-DTPA renal scans to about a tenth of the original effort. Furthermore, the accuracy of our DL model appears to be robust to the GFR level (as shown in the Bland-Altman plots in Fig. 6) and the presence of renal mass based on our subgroup analysis, unless they are extremely large. In fact, our DL model efficiently processed 24 364 DTPA scans in less than a second for each. While ^99m^Tc-DTPA scans are popularly used for GFR measurement, they are known to have limited precision with relatively large variability [24, 25]. The potential sources of this variability can be broadly categorised into two major components: (1) the image acquisition process, and (2) the image analysis process. Regarding the first component, there is no currently available literature investigating the test-retest reliability of ^99m^Tc-DTPA renal scans in the same patients. Nevertheless, the application of our DL model could minimise human error and variability in the image analysis process, thereby enhancing precision.

A unique aspect of our research is the use of existing ROI labels from standard reports already stored in our PACS, which avoided the laborious task of generating large amounts of labels, a common challenge in DL training. We extracted the ROIs using a colour-based algorithm and assured their accuracy by comparing kidney counts in report form with those calculated from the extracted ROIs. Our approach is fully automated, from downloading the DICOM files to processing the images, and can serve as a model for large-scale DL studies dealing with ROIs in related fields. Of note, our DL model tended to slightly underestimate GFR in the right kidney as indicated in Fig. 5a (slope = 0.982). We speculated that this might be due to the proximity of the liver to the right kidney, unlike the left kidney, potentially leading our DL model to set a tighter ROI for the right kidney to avoid including non-specific activity from the liver. Notably, the recent study by Pi et al. [26] differs from our approach in that they applied CNN regression to directly derive kidney counts or GFR values from dynamic renal images instead of using a DL to generate ROIs. Comparing our result of a CCC of 0.978 with the reported *R*^2^ values of 0.93 (predicting the kidney count, and then calculating GFR by the Gates method) and 0.85 (predicting GFR) in their study for total GFR, we speculate that generating the ROI itself may allow more accurate measurement of GFR than directly predicting the kidney count or GFR. The GFR values derived from our DL model are explainable and can be corrected by modifying the ROI if necessary. In addition, our model for setting the ROIs for kidneys and corresponding background can be used not only for GFR measurement, but also for generating renograms to evaluate renal obstruction or renovascular hypertension. Another previous study concentrated on assessing GFR from ^99m^Tc-DTPA renal SPECT/CT imaging, incorporating DL techniques for the segmentation of kidneys on CT scans [13]. This study predominantly adopted a 2D U-Net architecture to delineate the kidneys on the CT images associated with the ^99m^Tc-DTPA renal SPECT scans. By contrast, our research utilises a 2D U-Net architecture for segmenting the kidneys, while distinctively making use of renal scans and pre-established ROIs stored within our PACS system. This method is particularly relevant because renal scans are frequently acquired without accompanying CT scans. Consequently, our methodology is more closely aligned with the standard practices within the field, thereby potentially increasing its utility and significance in clinical environments.

Our study has some limitations, such as being conducted at a single institution, which may affect the broader applicability of the findings. However, the use of a 12-year dataset and the inclusion of various gamma cameras and multiple operators for analysis may partly mitigate this concern. Second, we excluded cases with severely reduced renal function. This could limit the application of our DL model in clinical scenarios when renal function is severely diminished. However, in most cases, kidneys with severely decreased function are not visually distinguishable even by experienced technologists, or can only be manually segmented by referring to other corresponding images such as CT images. If such cases and ROI labels were also to be included in the training set, the overall performance could possibly decrease. Generally, in deep-learning training, the use of a carefully selected dataset is preferred over a large unrefined dataset. Further study is warranted to test the applicability of such DL model for kidneys with severely decreased function. Third, our DL model gave inconsistent results as to whether tumours were included in the ROI, as shown in the representative Fig. 7. Although ^99m^Tc-DTPA scans are commonly used for the preoperative assessment of split renal function, there is no clear statement on whether or not to include the tumour when calculating GFR. On a planar renal scan, it is not possible to perfectly separate tumour from functional renal parenchyma. Finally, while the DL-based ROI generation method could be more reproducible than manual methods, our study has not directly demonstrated its superior reproducibility. Therefore, further research is warranted to evaluate this issue.

## Conclusions

Our 2D-CNN model with U-Net architecture demonstrated excellent performance in the segmentation of kidney ROIs on ^99m^Tc-DTPA scans. The GFR values from automatically generated ROIs showed high concordance with those from ROIs manually drawn by experts. The DL can simply complete GFR measurements without any additional effort for manual calibration in 80–90% of patients, with absolute differences of less than 5 mL/min/1.73 m^2^ or relative differences within 10%. Therefore, our research may allow simple operator-independent analysis of ^99m^Tc-DTPA scans for GFR measurement in a reproducible manner.

### Electronic supplementary material

Below is the link to the electronic supplementary material.

Supplementary Material 1

Supplementary Material 2

Supplementary Material 3

Supplementary Material 4

## Data Availability

The datasets generated and analysed during the current study are available from the corresponding author on reasonable request.
