# Supplementary figures and images for: Deep learning-based measurement of split glomerular filtration rate with 99mTc-diethylenetriamine pentaacetic acid renal scan
Source: EJNMMI Phys. 2024 Jul 17;11:64. doi: 10.1186/s40658-024-00664-w (PMC11254887; doi:10.1186/s40658-024-00664-w)

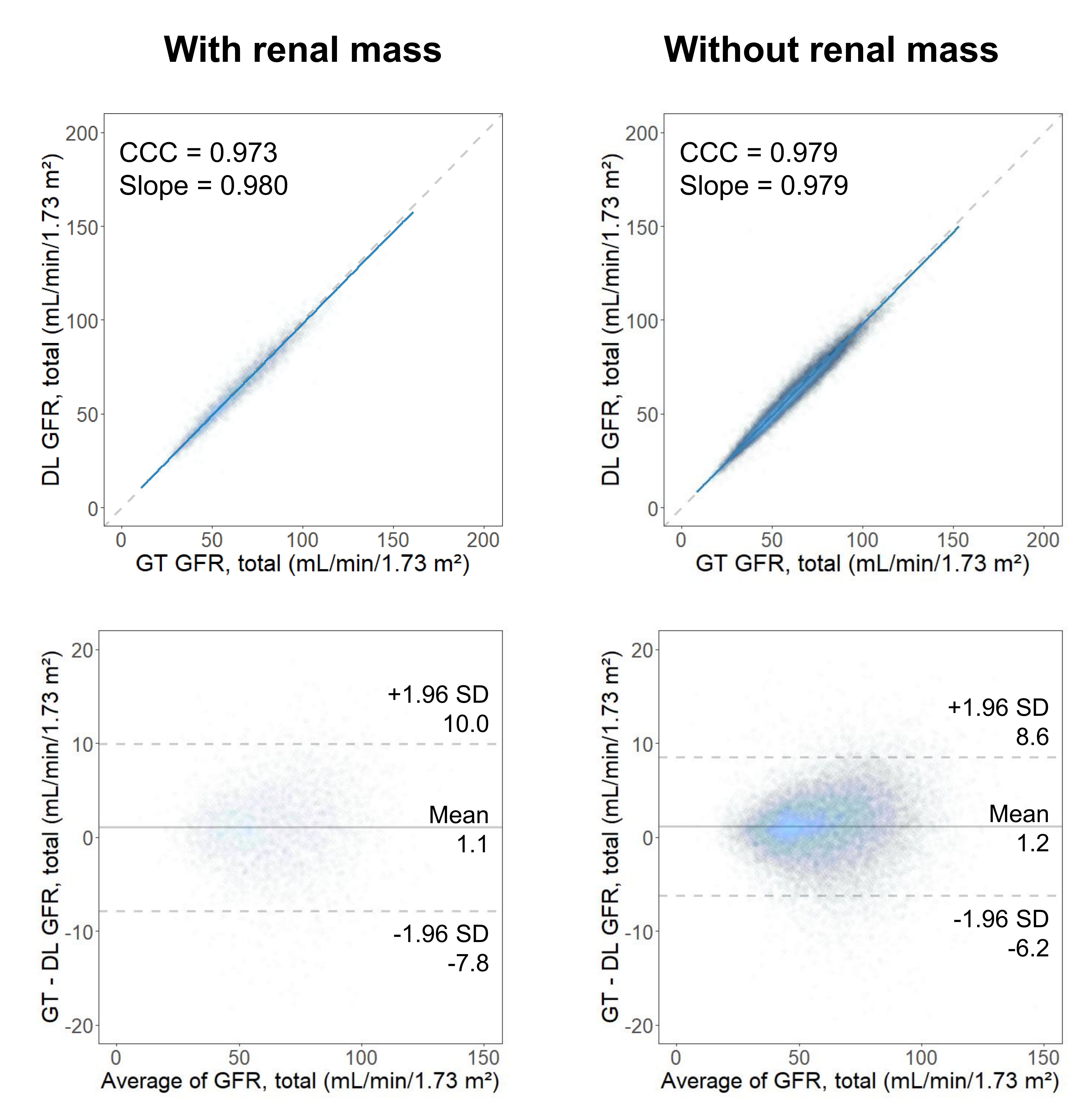

Supplement: Supplementary file 3 — Supplementary Material 3 [file 40658_2024_664_MOESM3_ESM.tif]
