# Supplementary material for: Deep learning-based measurement of split glomerular filtration rate with 99mTc-diethylenetriamine pentaacetic acid renal scan
Source: EJNMMI Phys. 2024 Jul 17;11:64. doi: 10.1186/s40658-024-00664-w (PMC11254887; doi:10.1186/s40658-024-00664-w)
Supplement: Supplementary file 4 — Supplementary Material 4 [file 40658_2024_664_MOESM4_ESM.docx]

**Supplementary Table 1.** The number of scans performed using each gamma camera

| **Cameras** | **Number of Scans** |
| --- | --- |
| Intevo Bold | 80 |
| Intevo 16 | 39 |
| Symbia E | 11,536 |
| Symbia E2 | 3,350 |
| Symbia T2 | 2,083 |
| E.cam | 5,417 |
| Evo Excel | 1,859 |
| **Total** | **24,364** |

**Supplementary Table 2.** Summary of ^99m^Tc-DTPA scan sessions per patient.

| Sessions per patient | Patients (*n*) | Total Scans (*n*) | Time interval between scans (months) |
| --- | --- | --- | --- |
| 1 | 8,743 | 8,743 | N/A |
| 2 | 1,329 | 2,658 | 5.9 (IQR 4.4–12.4) |
| 3 | 1,056 | 3,168 | 7.5 (IQR 4.9–13.1) |
| 4 | 542 | 2,168 | 12.1 (IQR 5.8–18.6) |
| 5 | 289 | 1,445 | 11.9 (IQR 6.4–12.9) |
| 6 | 263 | 1,578 | 12.1 (IQR 6.8–12.9) |
| 7 | 300 | 2,100 | 12.1 (IQR 6.8–12.6) |
| 8 | 221 | 1,768 | 12.1 (IQR 6.8–12.6) |
| 9 | 56 | 504 | 12.1 (IQR 6.8–12.9) |
| 10 | 21 | 210 | 12.0 (IQR 6.5–12.6) |
| 11 | 2 | 22 | 11.8 (IQR 7.1–12.4) |
| Total | 12,822 | 24,364 |  |

Data are expressed as number or median (interquartile range [IQR])
